# Socio-Economic Inequalities in Survival of Patients with Prostate Cancer: Role of Age and Gleason Grade at Diagnosis

**DOI:** 10.1371/journal.pone.0056184

**Published:** 2013-02-13

**Authors:** Kashif Shafique, David S. Morrison

**Affiliations:** 1 Institute of Health and Wellbeing, College of Medical, Veterinary and Life Sciences, University of Glasgow, Glasgow, United Kingdom; 2 Department of Community Medicine, Dow Medical College, Dow University of Health Sciences, Karachi, Pakistan; 3 West of Scotland Cancer Surveillance Unit, Glasgow, United Kingdom; Vanderbilt University, United States of America

## Abstract

In the United Kingdom, survival of prostate cancer patients has improved since the 1990s. A deprivation gap in survival (better survival for the least deprived compared with the most deprived) has been reported but it is not known if differential distribution of earlier age or lower grade disease at diagnosis might explain such patterns. We therefore investigated the impact of age and Gleason grade at diagnosis on the deprivation gap in survival of prostate cancer patients over time. Incident cases of prostate cancer (ICD-10 C61) from the West of Scotland were extracted from the Scottish Cancer Registry from 1991 to 2007. Socio-economic circumstances were measured using the Scottish Index for Multiple Deprivation 2004 (SIMD). Age and deprivation specific mortality rates were obtained from the General Registrar Office for Scotland (GRO(S)). The survival gradient across the five deprivation categories was estimated with linear regression, weighted by the variance of the relative survival estimate. We examined the data for 15,292 adults diagnosed with prostate cancer between 1991 and 2007. Despite substantial improvements in survival of prostate cancer patients, a deprivation gap persists throughout the three periods of diagnoses. The deprivation gap in five year relative survival widened from −4.76 in 1991–1996 to −10.08 in 2003–2007. On age and grade-specific analyses, a significant deprivation gap in five year survival existed between all age groups except among patients' age ≥75 and both low and high grade disease. On multivariate analyses, deprivation was significantly associated with increased excess risk of death (RER 1.48, 95% CI 1.31–1.68, p-value<0.001) independent of age, Gleason grade and period of diagnosis. The deprivation gap in survival from prostate cancer cannot be wholly explained by socio-economic differentials in early detection of disease. Further research is needed to understand whether differences in comorbidities or treatment explain inequalities in prostate cancer outcomes.

## Introduction

Survival of patients with prostate cancer has improved significantly since the 1990s in many Western countries. Improvements in survival have been attributed to greater detection of early, localised disease and advances in treatment. Despite substantial improvements in survival of patients with prostate cancer, however, significant disparities in survival have been observed between socio-economic and racial groups [1], [2].

In the UK, a deprivation gap in survival of men with prostate cancer (better survival for the least deprived compared with the most deprived) has been reported previously in studies carried out in England, Wales and Scotland [3], [4]. The deprivation gap in five-year survival from prostate cancer in England and Wales increased from −1.2% (1.2% worse survival in the most deprived patients as compared to the least deprived) in 1986–1990 to −7.2% in 1996–1999 [3]. In Scotland, although the relative survival of prostate cancer patients improved 11% on average every five years during the period 1986–2000 [4], the deprivation gap has also increased and the difference in relative survival between the least deprived and the most deprived was similar to that in other parts of the UK, at −6.9% during the period 1996–2000 [4].

In the United States, both socio-economic and racial inequalities in survival have been attributed to delayed diagnosis and less aggressive treatment [2], [5]. Choice of treatment may also have a substantial role in the observed survival trends as after a diagnosis of prostate cancer men from low socio-economic groups are substantially less likely to be treated with radical surgery or radiotherapy [6]. It has also been suggested that socio-economic survival differences may be explained by differences in age at presentation; variations in aggressiveness and stage of disease at the time of diagnosis; or by inequalities in access to health care services between socio-economic groups [4]. There are important practical differences in addressing each explanation, ranging from better public health promotion to reconfiguration of health services to ensure equity in referral and access to specialist services. However, the effect of these factors on the observed deprivation gap in survival remains unclear and not well researched.

The aim of our study was to examine the overall and deprivation-specific survival trends for men with prostate cancer in the West of Scotland and to determine whether age or Gleason grade at diagnosis explained any difference in survival between socio-economic groups.

## Materials and Methods

### Incidence data

We examined the data of adults (aged 15–100 years) diagnosed with a first, primary (excluding non-melanoma skin cancer) malignant neoplasm of the prostate during 1991–2007 in the West of Scotland, using the International Classification of Diseases (ICD) 10 code C61 for prostate cancer. We used the data of all incident prostate cancer cases from 1^st^ January 1991 to 31^st^ December 2007 and followed-up till 31^st^ December 2008, as this was the most recent year of complete survival data available at the time of this analysis. Incidence data were linked to death records provided by the General Registrar Office for Scotland (GRO(S)). The vital status of all patients was considered to be known up to 31^st^ December 2008. Patients identified from death certificate records only were excluded (n = 225, 0.01% of all registered records) from this analysis. This is because they had zero survival due to having the same incidence and death dates. Thirty patients belonging to areas other than the West of Scotland and one patient older than 100 years were also excluded from this analysis.

Socio-economic status of individuals was assigned by matching their postcode of residence at diagnosis to the Scottish Index of Multiple Deprivation (SIMD) 2004 score. SIMD is an area-based measure of socio-economic circumstances that ranks small geographic areas of Scotland (datazones) from 1 (most deprived) to 6505 (least deprived) using 31 indicators that cover current income, employment, health, education, housing and access [7]. The datazones are further grouped into national quintiles that range from least deprived to the most deprived. The Gleason grading system is known to be associated with prostatic cancer prognosis [8] and was used to describe tumour morphology. Gleason grade was extracted from the Scottish Cancer Registry where available.

### Ethical approval

Permission to use Scottish Cancer Registry data was given by the Privacy Advisory Committee of the Information Services Division of National Health Service National Services Scotland. All relevant Caldicott Guardians granted permission for use of their Cancer Registry data.

### Statistical analysis

For relative survival analysis, we compared the survival in prostate cancer patients with men of the same age, calendar year and socio-economic group in Scotland.

Population life tables were obtained for the years 1991–2007 and we assumed that age and deprivation-specific survival was the same in 2008 as 2007, because life tables for 2008 were not available. We used all Ederer I, Ederer II and Hakulinen methods to relative survival, as the results were identical with all three methods so we presented the results of Ederer II method.

Age at diagnosis was categorised in three groups as <65, 65–74 and ≥75 years. To explore the time trends in survival, year of diagnosis was categorised into three categories as 1991–1996, 1997–2002 and 2003–2007. The rationale behind using these categories of year of diagnosis was that Scottish Cancer Registry started to record Gleason grade from 1^st^ January 1997. Therefore, by making these categories, grade specific analysis could be carried out on all subjects in the two later periods. Gleason grade was categorised as low (Gleason 2–6), intermediate (Gleason = 7) and high grades (Gleason 8–10).

We estimated one, three and five year relative survival rates for prostate cancer patients diagnosed in the West of Scotland by age, deprivation quintile, Gleason grade and calendar period of diagnosis. Relative survival is the ratio of the observed (absolute) survival of prostate cancer patients and the survival that would have been expected if the patient had had the same age and deprivation specific mortality in each period (background population mortality); a technique which has been used earlier in the estimation of the deprivation gap in Scotland [4]. Survival probabilities for cancer patients were estimated at 6 month intervals from diagnosis to 5 years. Cumulative relative survival up to 5 years after diagnosis was estimated for patients diagnosed in calendar periods 1991–1996, 1997–2002 and 2003–2007.

Both cohort and complete approaches were used to estimate observed survival. In the estimation of five year survival using the cohort approach, all patients must have a potential follow-up of at least five years. With the complete approach recently diagnosed patients with less than 5 years follow-up can also be included in the analysis along with those with potential follow-up of at least 5 years. Both techniques provided similar results and the complete approach survival analyses are presented in this study.

Survival gradients across the five deprivation quintiles based on SIMD score were estimated with linear regression, weighted by the variance of the relative survival estimate [9] using STATA software (StataCorp, version 11). The difference between the relative survival rates fitted by the linear regression model for the least deprived and the most deprived categories is presented as the “deprivation gap” in survival. The deprivation gap is reported as negative (−) if the most deprived group has lower survival than the least deprived. Average changes in the deprivation gap between the three periods have also been reported, taking into account the shorter duration of the final period (1 year shorter than the two earlier periods).

To investigate the impact of age and Gleason grade of tumour on the deprivation gap, the gap was estimated stratifying by age and Gleason grade categories. The deprivation gap was estimated by least-squares linear regression, weighted by the variance of each of the relative survival estimate stratified by age and Gleason grade. The significance was evaluated with a likelihood ratio test at the 5% level.

To investigate the major determinants associated with mortality, a full likelihood approach was used to model the excess mortality [10]. This method estimates the excess risk of mortality associated with prostate cancer as mortality of prostate cancer patients is compared with a matched cohort using the background population mortality. Age at diagnosis, Gleason grade, deprivation and period of diagnosis were used as independent variables in modelling excess risk of death. We analysed the effects of baseline variables on survival using Cox proportional hazards models [11], [12].

## Results

A total of 15,519 men were identified, who were registered with a diagnosis of prostate cancer in the West of Scotland from 1991–2007. Details of excluded cases are provided in table 1, a total of 15,292 patients diagnosed in the West of Scotland were included in the final analysis. The proportion of men younger than 65 years at diagnosis increased over the study period, from 15.4% between the years 1991–1996 to 23.6% between the years 2003–2007 (*X* ^2^, p<0.001). The highest proportion of cases were observed in older age groups, 39.4% of cases (n = 6,023) occurred in 65–75 years of age and 41.1% of cases (n = 6,285) in men older than 75 years. Mean age at incidence decreased during the same periods from 73.2±8.74 to 71.1±9.08. Overall, 17.2% of patients were in the least deprived group while 27.5% were in the most deprived group. Baseline characteristics of the study population are described in table 1.

**Table 1:** Baseline characteristics of prostate cancer patients registered in the West of Scotland from 1991–2007.

	Total patients		All deaths	
	n	%	n	%
**Total registered cases**	15,549	_	9,355	60.2
Patients not residing in the West of Scotland	30	_	19	63.3
Zero survival or death certificate only (DCO)	226	_	_	_
Age more than 100 years at diagnosis	1	_	1	100.0
Patients included in final analysis	15,292		9,109	59.7
**Age at incidence (years)**				
Age <65	2,984	19.5	1,086	11.9
Age 65–74	6,023	39.4	3,232	35.5
Age ≥75	6,285	41.1	4,791	52.6
**Gleason Grade**				
Gleason <7	4,065	37.2	1,421	27.1
Gleason = 7	2,231	20.4	756	14.4
Gleason 8–10	3,311	30.3	2,051	39.0
Unknown Gleason	1,316	12.1	1,026	19.5
**SIMD 2004, Quintiles**				
1 (least deprived)	2,623	17.2	1,295	14.2
2	2,278	14.9	1,219	13.4
3	2,450	16.0	1,444	15.9
4	3,737	24.4	2,365	26.0
5 (most deprived)	4,202	27.5	2,786	30.6
**Period of Diagnosis**				
1991–1996	4,369	28.6	3,855	42.3
1997–2002	5,474	35.8	3,580	39.3
2003–2007	5,449	35.6	1,674	18.4

Period of diagnosis was based on incidence date recorded in cancer registry.

Regarding disease grade, more than half of the men (57.6%) had either low grade (Gleason <7) or intermediate grade disease (Gleason = 7). Proportions of low grade disease significantly increased during the study period while the proportion of unknown grade patients significantly reduced from 16.7 in 1997–2002 to 7.7% in 2003–2007 (*X* ^2^, p<0.001).

Both short and long term survival of prostate cancer patients have improved since 1991 (table 2). Relative survival at 1 year increased significantly from 83.2% in 1991–1996 to 92.1% in 2003–2007 (fitted, deprivation adjusted increase of 4.5% between periods). Five year survival increased from 58.2% to 78.6% in men over the same period, an average deprivation adjusted increase of 10.2% between six year periods. While five year survival increased between both latter periods, there was a larger increase between the first two periods from 1991–1996 to 1997–2002 (11.8%) and a relatively smaller increase (7.6%) in survival between the two later periods (Table 2).

**Table 2:** Trends in relative survival (%) of prostate cancer patients by period of diagnosis in the West of Scotland: 1991 to 2007.

	Calendar period of diagnosis						Average change (%)	
Time since diagnosis	1991–1996		1997–2002		2003–2007		between periods^a^	
	Survival, %	(95% CI)	Survival, %	(95% CI)	Survival, %	(95% CI)		(95% CI)
1 year	83.2	(81.7, 84.5)	90.9	(89.9, 91.9)	92.1	(91.2, 93.0)	4.5	(−19.4,28.3)
3 years	67.4	(65.5, 69.2)	78.1	(76.5, 79.6)	83.1	(81.6, 84.6)	7.8	(−13.1, 28.8)
5 years	58.2	(56.0, 60.3)	71.0	(69.1, 72.8)	78.6	(76.4, 80.8)	10.2	(−8.9, 29.3)

a = Mean absolute change in relative survival between periods adjusted for deprivation.

**Figure 1:**
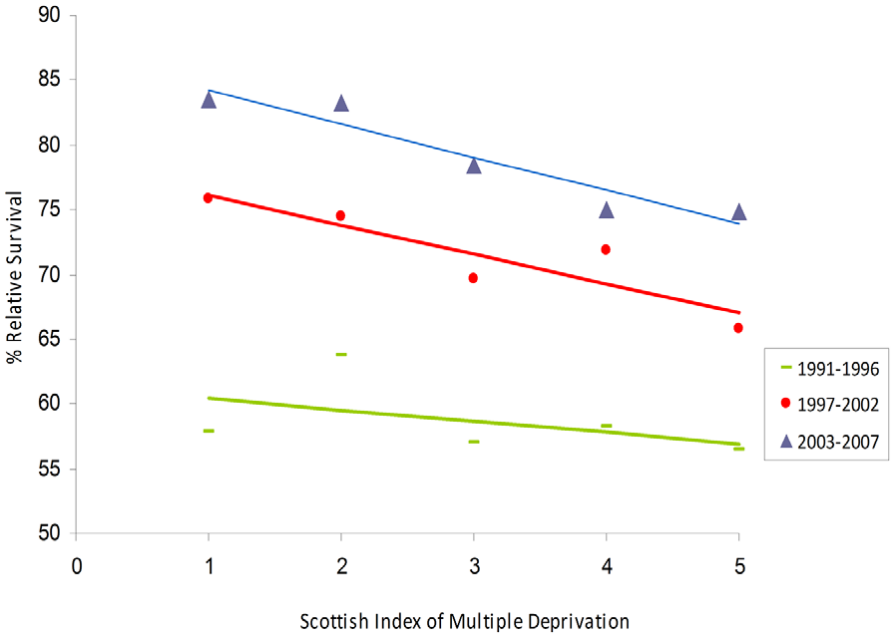
Deprivation gap in 5-year relative survival from prostate cancer from 1991–2007 in the West of Scotland.

Despite substantial improvements in survival of prostate cancer patients, there was a deprivation gap in each of the three periods of diagnoses (Figure 1). Large improvements in survival occurred over time in all socio-economic groups. The deprivation gap was smaller in 1991–1996 and widened during later periods, due to larger improvements in survival among the least deprived group. There was little change in the deprivation gap in one year and three year survival over time but the deprivation gap in five year survival increased to a greater extent, from −4.76 in 1991–1996 to −9.08 in 1996–2002, with a relatively small change to the most recent period (table 3).

**Table 3:** Trends in the deprivation gap in relative survival of prostate cancer patients by time since diagnosis and calendar period in the West of Scotland during 1991–2007.

	Calendar period of diagnosis^a^						Average change (%)	
Time since diagnosis	1991–1996		1997–2002		2003–2007		between periods^b^	
	Deprivation gap (%)	(95% CI)	Deprivation gap (%)	(95% CI)	Deprivation gap (%)	(95% CI)	Deprivation gap (%)	(95% CI)
1 year	−4.68	(−7.17, −2.19)	−4.52	(−6.02,−3.02)	−3.96	(−5.7, −2.18)	0.19	(−0.30,0.41)
3 year	−6.72	(−13.21, −0.23)	−8.08	(−12.65, −3.50)	−7.56	(−10.05, −5.07)	−0.42	(−7.31, 6.47)
5 year	−4.76	(−10.55, 1.03)	−9.08	(−12.37, −5.78)	−10.08	(−13.05, −7.11)	−2.65	(−14.83, 23.65)

a = Relative Survival estimated by complete approach,

b = Mean absolute change in survival in between periods adjusted for deprivation.

Further analysis was carried out to investigate the impact of age and Gleason grade at diagnosis on the deprivation gap in survival. For men younger than 65 years the deprivation gap was significant during the two earlier study periods, 1991–2002. Conversely, for patients aged 65 years and older, no significant deprivation gap was identified between 1991 and 2002 but a significant gap appeared in the most recent period, 2003–07. This was −9.9% in men aged 65–74 and −7.4% in men aged ≥75 years. Grade-specific analysis from 1997 onwards (when information on grade became available) showed a significant deprivation gap in five year survival of −7% and −9% in men with low grade (Gleason 2–6) and high grade disease (Gleason 8–10), respectively. There was no significant deprivation gap for men with intermediate grade disease (Gleason = 7) (table 4). A significant deprivation gap of −9% appeared in 2003–07 for patients with unknown Gleason grade.

**Table 4:** Deprivation gap in 5-year relative survival of prostate cancer patients by age, Gleason grade and calendar period in the West of Scotland during 1991–2007.

	Calendar period of diagnosis^a^					
Characteristics	1991–1996		1997–2002		2003–2007	
	Deprivation gap (%)	(95% CI)	Deprivation gap (%)	(95% CI)	Deprivation gap (%)	(95% CI)
Age <65	−13.6	(−23.24, −3.95)	−13.32	(−16.23,−10.41)	−7.72	(−24.49, 9.05)
Age 65–74	−5.36	(−16.28, 5.56)	−11.48	(−13.20, 9.76)	−9.92	(−12.11, −7.73)
Age ≥75	2.00	(−1.65, 5.66)	−1.44	(−12.22, 9.34)	−7.36	(−12.32, −2.40)
Gleason <7	_	_	−6.64	(−11.82,−1.46)	−6.88	(−12.95, −0.80)
Gleason = 7	_	_	−0.4	(−7.16, 6.36)	3.92	(−6.47, 14.31)
Gleason 8–10	_	_	−10.12	(−16.63, −3.61)	−8.76	(−20.37,2.85)
Unknown Gleason	_	_	−1.8	(−14.75,11.14)	−8.76	(−13.72,−3.80)

a = Relative Survival estimated by complete approach.

Deprivation was associated with increased risk of death, independent of age at incidence and period of diagnosis, for both low grade (age-adjusted RER for most deprived compared with least deprived = 2.61, 95% CI 1.09–6.26, p-value<0.001) and high grade disease (RER = 1.36, 95% CI 1.10–1.69, p-value<0.005) groups. No deprivation effect on risk of death was observed for intermediate grade disease. During the later period (2003–2007), an 81% reduction in the risk of death was observed in low grade disease group, 61% reduction in the intermediate group and 22% risk reduction in the high grade group (table 5). The risk of death increased by 87% among those with unknown grade in the later period compared to the earlier one.

**Table 5:** Grade specific risk of excess mortality due to prostate cancer modelled using the full likelihood approach.

	Gleason <7				Gleason = 7				Gleason 8–10				Unknown Gleason			
	Relative Excess Risk (95% CI)			p value	Relative Excess Risk (95% CI)			p value	Relative Excess Risk (95% CI)			p value	Relative Excess Risk (95% CI)			p value
*SIMD 2004, Quintiles*																
1 (least deprived)		1				1				1				1		
2	1.20	(0.41,	3.52)	0.742	0.94	(0.44,	1.99)	0.871	1.05	(0.81,	1.36)	0.702	1.25	(0.90,	1.73)	0.192
3	1.69	(0.62,	4.64)	0.309	1.01	(0.48,	2.12)	0.981	1.42	(1.13,	1.79)	0.003	1.02	(0.73,	1.46)	0.875
4	1.61	(0.61,	4.26)	0.005	1.36	(0.73,	2.55)	0.331	1.48	(1.19,	1.84)	<0.001	1.06	(0.78,	1.43)	0.722
5 (most deprived)	2.61	(1.09,	6.26)	0.031	0.84	(0.39,	1.82)	0.657	1.36	(1.10,	1.69)	0.005	1.47	(1.13,	1.92)	0.004
*Period of diagnosis*																
1997–2002		1				1				1				1		
2003–2007	0.19	(0.05,	0.74)	0.017	0.39	(0.27,	0.68)	<0.001	0.78	(0.68,	0.90)	<0.001	1.87	(1.55,	2.40)	<0.001

All estimates were adjusted for age at incidence.

## Discussion

This study confirms that survival of patients with prostate cancer has significantly improved in the West of Scotland since 1991. Despite this, socio-economic inequalities in survival of prostate cancer patients increased in the most recent period of observation. Socio-economic inequalities in survival were of a similar magnitude at different ages and Gleason grades, suggesting that the effects of earlier detection of prostate cancer – such as by greater PSA testing - in less deprived populations are unlikely to wholly explain them.

### Temporal trends in survival

Improvements have been observed in both short term (1-year) and long term survival (5-year) in the West of Scotland. Overall, improvement in survival are consistent with reports from England and Wales [3]. Survival of patients with prostate cancer has improved during corresponding periods in many European countries. Denmark for example, observed approximately 7% improvement in five year survival between 1985–2004 [13]. Another recent report from Denmark also suggested similar findings [14]. In the Netherlands, survival improved by approximately 1.8% annually from 1989 to 2006 [15].

Disease related factors are affected by PSA testing, which can partly explain both the increase in incidence and survival. The introduction of PSA testing may have led to stage and grade migration of prostate cancer [16]. This has resulted in a rapid shift in the biological spectrum of the disease such that at a population level prostate cancer is not necessarily the fatal and incurable disease it once was. A higher proportion of individuals are now diagnosed with localized, small volume and low grade prostate tumours[17], which are treated aggressively and, ultimately, lead to better survival.

In recent years, greater awareness among general practitioners is another factor that might have contributed to better management of patients. For instance, greater awareness of prostate cancer may have increased demands for PSA testing by at younger age men or family members of men diagnosed with prostate cancer leading to higher detection [18]. More intensive monitoring of patients after diagnosis could also have played a role in better survival and lower mortality. Besides the possible benefits associated with PSA testing, there is also a risk of over diagnosis and treatment of asymptomatic cases of prostate cancer which might have never manifested as clinically symptomatic disease during their lifetime [16].

Another potential factor contributing to improvement in survival is the improvement and advancement in treatment. The effect of treatment *per se* on improved survival in this population is difficult to quantify accurately with available data, however there is some evidence from other regions that therapeutic treatment has contributed in some way to the observed survival improvements [19].

### Socio-economic inequalities in survival

Despite the overall improvement in survival of prostate cancer patients, socio-economic inequalities in survival of prostate cancer patients remained persistent slightly increased over the periods 1997–2002 and 2003–2007. Estimates of one, three and five year deprivation gaps in survival have been presented along with the trends of these gaps over time. The concurrent increase in survival differences between the least deprived and the most deprived men, taken with the more rapid increase in incidence of low grade disease among the least deprived group may suggests that individuals from higher socio-economic groups had greater access to PSA testing during these recent periods [17]. The deprivation gap in survival for prostate cancer patients has been widening as reported earlier from Scottish national data [4].

Socio-economic differences in survival have been observed in many countries including Australia [20], New Zealand [21], England and Wales [3] and the USA [5]. In England and Wales, recent improvements in survival of many adult cancers including prostate has been more marked for the least deprived groups compared with the most deprived [9]. One of the major aims of the NHS Cancer Plan, implemented in 2000 in England and Wales, was to tackle inequalities in cancer survival. However, a recent study examined the survival differences among affluent and deprived cancer patients before and after the plan and reported that there is very little variation in the deprivation gap in the 10 years after the implementation of the NHS Cancer Plan [22].

Several factors can contribute towards lower survival among more deprived men including older age, advanced stage and aggressive disease at presentation, higher prevalence of comorbidities and unhealthy lifestyle habits. The pattern of prostate cancer presentation among deprived men characterized by lower uptake of PSA and late diagnosis with advanced stage disease has not changed over the years [23]. Whether the lower testing rate is due to the lack of availability of the test from the General Practitioners or the men in more deprived groups do not simply come forward for testing need to be further investigated.

Some studies have attributed the socio-economic inequalities in survival to higher levels of comorbidity among more deprived patients (Clarke, 2008). Along with comorbidity, there are numerous other factors that can contribute to socio-economic inequalities in survival. For example, risk behaviours such as heavy smoking or alcohol intake, which are more prevalent among the most deprived patients, could explain some of the observed differences in [24], [25].

Although plausible, such explanations may not be consistent with our findings. Age and deprivation specific background population mortality rates were used to estimate relative survival adjusted for differentials in comorbidity between socio-economic groups. Differential comorbidity can only contribute to socio-economic inequalities in relative survival only if they produce additional disease-specific effects, such as altering response to treatment [26]. Comorbidity can partly explain why non-metastatic prostate cancer patients in England from poorer socio-economic backgrounds were less likely to receive aggressive surgical or radiological treatment compared with the least deprived population group [27] . If differential co-morbidity between these groups of prostate cancer patients explains persistent or widening deprivation gap in survival, it would imply that the impact of comorbidity on treatment (or outcome) increased more among the most deprived than the least deprived [26]. Since our findings show that cancer survival improved more rapidly for the least deprived groups, socio-economic differences in the diagnosis of prostate cancer and/or treatment of the disease rather than comorbidity may be the more plausible potential explanation of survival differences between social groups.

### Age, Gleason grade at diagnosis and deprivation gap in survival

Age and Gleason grade are considered to be strong predictors of prognosis of prostate cancer patients. Large improvements in survival were observed among those diagnosed with low grade disease (Gleason <7). The risk of death significantly reduced among men with low grade disease in the most recent period (2003–2007) compared with earlier period (1997–2002). A reduction of 80% in the excess risk of death in the most recent period, suggests that the biological spectrum of prostate cancer has changed over time and in more recent years, we are treating an entirely new disease which is perhaps a very low risk cancer. Despite the largest improvement in survival in this disease group, more than double the risk of death was observed for the most deprived group compared with the least deprived.

In this dataset, least deprived were more likely to be diagnosed at a younger age and with lower grade. Age and Gleason grade-specific analysis show some reduction in deprivation gap in survival among younger men. However, there were no significant changes in size of deprivation gap within both low and high grade disease groups. This suggests that there may be disparities among different socio-economic groups other than the difference in age and disease grade at presentation.

Stage at diagnosis is also an important factor associated with the survival of prostate cancer patients [24]. For example in the US, Black men have poorer survival compared to Whites and one of the explanations for that is the relatively late presentation by Black men with advanced and metastatic disease at diagnosis. Delayed presentation by low socio-economic groups is also considered a contributory factor in socio-economic inequalities in cancer survival in the UK. However, the hard evidence on this for prostate cancer in the UK is still sparse. There is some evidence from recent studies investigating the impact of race in clinical presentation of prostate cancer in UK, but no significant differences were observed between White and Black men's age, disease grade and stage at presentation [28], [29]. Unfortunately, disease stage information was not present in the Scottish Cancer Registry data, so the role of disease stage in the existing deprivation gap in survival could not be investigated.

### Strengths and limitations of this research

Scotland Cancer Registry data for the West of Scotland was used for this analysis providing a fairly large sample with long follow-up of patients to carry out this analysis. Different techniques were used to estimate the relative survival, i.e. cohort and complete approach and both produced identical results. Those whose prostate cancer diagnosis was only made after death or who died on the day of diagnosis were excluded from this analysis. Such patients comprised a small proportion of the study population (n = 226, 0.01%). Exclusion of these cases from the analysis does not explain the existing and widening deprivation gap in relative survival for multiple reasons. First, there was no significant difference in distribution of these cases between socio-economic groups. Secondly, the proportion of patients excluded from the analysis is very small to have any significant impact on the estimates provided in this analysis.

The SIMD was used as a proxy measure of socio-economic circumstances of prostate cancer patients in the present analysis. As with all the area-based measures of deprivation, it assumes that all individuals living in a given geographical area experience the same level of deprivation and other associated factors. Individual level data such as level of education, occupation and income can provide stronger evidence of socio-economic circumstances; however, these data are not routinely collected in cancer registries. Earlier studies carried out in different regions of the UK, despite the use of different area-based measures, also provided similar results that the least deprived have better survival compared with the most deprived, which suggests that these findings are not just due to the measurement index. In addition, race has been widely used as a measure of socio-economic circumstances in the US and provided quite similar results suggesting that individuals from poorer background experience worse survival. Another important limitation is the unavailability of information on the stage of the cancer and on comorbidities in the Scottish Cancer Registry, both of which are well known prognostic factors in survival of patients.

## Conclusion

The substantial improvements in survival from prostate cancer are encouraging but persisting socio-economic inequalities remain. These seem unlikely to be explained by greater use of PSA to detect early prostate cancer in more affluent populations. Further research is needed to explore the interactions of differentials in comorbidities and treatment choice for prostate cancer in persistent or widening deprivation gap in prostate cancer survival.
